# 3-(Adamantan-1-yl)-1-[(4-benzyl­piperazin-1-yl)meth­yl]-4-ethyl-1*H*-1,2,4-triazole-5(4*H*)-thione

**DOI:** 10.1107/S1600536813031127

**Published:** 2013-11-23

**Authors:** Ebtehal S. Al-Abdullah, Hanaa M. Al-Tuwaijri, Ali A. El-Emam, C. S. Chidan Kumar, Hoong-Kun Fun

**Affiliations:** a Department of Pharmaceutical Chemistry, College of Pharmacy, King Saud University, PO Box 2457, Riaydh 11451, Saudi Arabia; b X-ray Crystallography Unit, School of Physics, Universiti Sains Malaysia, 11800 USM, Penang, Malaysia

## Abstract

In the title compound, C_26_H_37_N_5_S, the piperazine ring adopts a chair conformation with the exocyclic N—C bonds in pseudo-equatorial orientations. The piperazine ring (all atoms) subtends dihedral angles of 79.47 (9) and 73.07 (9)° with the triazole and benzene rings, respectively, resulting in an approximate U-shape for the mol­ecule. No significant inter­molecular inter­actions are observed in the crystal.

## Related literature

For the pharmacological properties of adamantane derivatives and adamantyl-1,2,4-triazoles, see: Vernier *et al.* (1969 ▶); El-Emam *et al.* (2004 ▶, 2013 ▶); Al-Deeb *et al.* (2006 ▶); Kadi *et al.* (2007 ▶, 2010 ▶). For related adamantyl-1,2,4-triazole structures, see: Al-Tamimi *et al.* (2010 ▶); Al-Abdullah *et al.* (2012 ▶); El-Emam *et al.* (2012 ▶). For the synthesis of the starting material, see El-Emam & Ibrahim (1991 ▶). For ring conformations and ring puckering analysis, see: Cremer & Pople (1975 ▶).
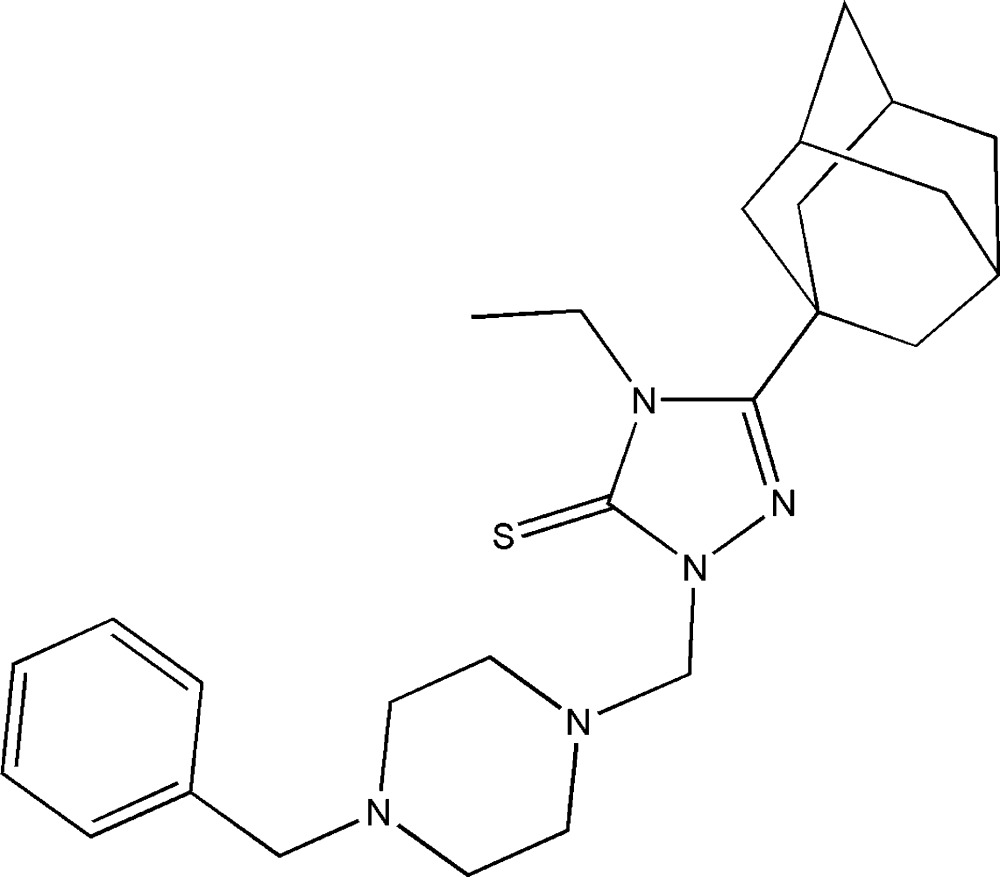


## Experimental

### 

#### Crystal data

C_26_H_37_N_5_S
*M* *_r_* = 451.67
Triclinic, 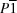

*a* = 10.5618 (2) Å
*b* = 11.2123 (2) Å
*c* = 11.3084 (2) Å
α = 89.974 (1)°
β = 77.619 (1)°
γ = 70.842 (1)°
*V* = 1232.03 (4) Å^3^
*Z* = 2
Cu *K*α radiation
μ = 1.33 mm^−1^
*T* = 296 K
0.69 × 0.51 × 0.39 mm

#### Data collection

Bruker APEXII CCD diffractometer
Absorption correction: multi-scan (*SADABS*; Bruker, 2009 ▶) *T*_min_ = 0.460, *T*_max_ = 0.626
13169 measured reflections
4319 independent reflections
3986 reflections with *I* > 2σ(*I*)
*R*_int_ = 0.025

#### Refinement

*R*[*F* ^2^ > 2σ(*F* ^2^)] = 0.040
*wR*(*F* ^2^) = 0.117
*S* = 1.08
4319 reflections
291 parameters
H-atom parameters constrained
Δρ_max_ = 0.22 e Å^−3^
Δρ_min_ = −0.19 e Å^−3^

### 

Data collection: *APEX2* (Bruker, 2009 ▶); cell refinement: *SAINT* (Bruker, 2009 ▶); data reduction: *SAINT*; program(s) used to solve structure: *SHELXTL* (Sheldrick, 2008 ▶); program(s) used to refine structure: *SHELXTL*; molecular graphics: *SHELXTL*; software used to prepare material for publication: *SHELXTL* and *PLATON* (Spek, 2009 ▶).

## Supplementary Material

Crystal structure: contains datablock(s) global, I. DOI: 10.1107/S1600536813031127/hb7161sup1.cif

Structure factors: contains datablock(s) I. DOI: 10.1107/S1600536813031127/hb7161Isup2.hkl

Supplementary material file. DOI: 10.1107/S1600536813031127/hb7161Isup3.cml

Additional supplementary materials:  crystallographic information; 3D view; checkCIF report

## Figures and Tables

**Fig. 1.**
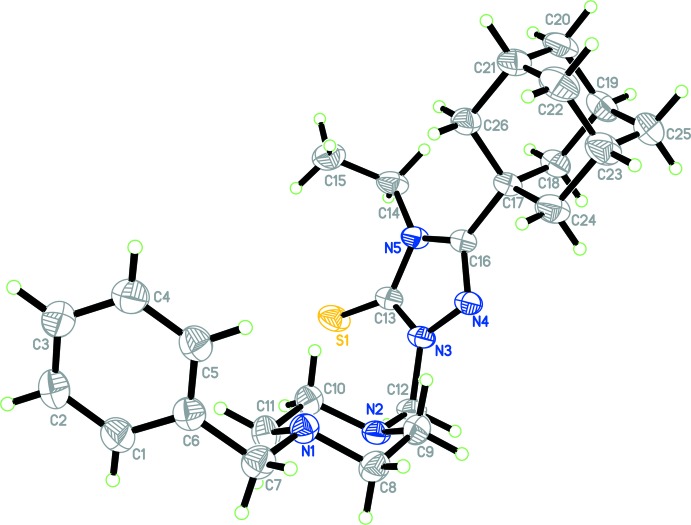
The molecular structure of the title compound with 30% probability displacement ellipsoids.

## supplementary crystallographic information

### 1. Comment

Adamantane derivatives were early recognized for their diverse biological activities including antiviral activity against the influenza (Vernier *et al.*, 1969) and HIV viruses (El-Emam *et al.*, 2004). In addition, adamantane derivatives were reported to exhibit marked antibacterial (Kadi *et al.*, 2007, 2010) and anti-inflammatory (El-Emam & Ibrahim, 1991) activities. In continuation of our interest in this area, we now describe the synthesis and structure of the title compound, (I).

The piperazine ring (N1/N2/C8–C11) ring, Fig. 1, adopts a chair conformation, with puckering parameters: Q = 0.5829 (17) Å, θ = 176.42 (17)°, and φ = 221 (3)° (Cremer & Pople, 1975) and a maximum deviation of 0.257 (1) Å at atom N1. The dihedral angle between the piperazine ring and the triazole (N3–N5/C13/C16) ring is 79.47 (9)°. The triazole (N3–N5/C13/C16) ring forms the dihedral angle of 53.07 (10)° with the benzene (C1–C6) ring. In the crystal, no significant intermolecular interactions beyond the expected packing contacts are observed.

### 2. Experimental

A mixture of 527 mg (2 mmol) of 3-(1-adamantyl)-4-ethyl-4*H*-1,2,4- triazole-5-thiol (El-Emam *et al.*, 1991), 1-benzylpiperazine (353 mg, 2 mmol) and 37% formaldehyde solution (1 ml), in ethanol (8 ml), was heated under reflux for 15 min until a clear solution was obtained. Stirring was continued for 12 h at room temperature and the mixture was allowed to stand overnight. Cold water (5 ml) was slowly added and the mixture was stirred for 20 min. The precipitated crude product were filtered, washed with water, dried, and crystallized from ethanol to yield 759 mg (84%) of the title compound (C_26_H_37_N_5_S) as crystals. *m.p.*: 441–443 K. Single crystals suitable for X-ray analysis were recrystallized from CHCl_3_:EtOH solution (1:1; 5 ml) through slow evaporation at room temperature.

^1^H NMR (CDCl_3_, 500.13 MHz): δ 1.13 (t, 3H, CH_2_CH_3_, J = 6.0 Hz), 1.68–1.73 (m, 6H, Adamantane-H), 1.98–2.10 (m, 9H, Adamantane-H), 2.72–2.76 (m, 4H, Piperazine-H), 3.24–3.26 (m, 4H, Piperazine-H), 3.72 (s, 2H, Benzylic-CH_2_), 4.15 (q, 2H, CH_2_CH_3_, J = 6.5 Hz), 5.02 (s, 2H, CH_2_), 7.20–7.54 (m, 5H, Ar—H). ^13^C NMR (CDCl_3_, 125.76 MHz): δ 12.74 (CH_2_CH_3_), 26.90, 34.36, 35.26, 38.82 (Adamantane-C), 40.62 (CH_2_CH_3_), 46.01, 50.37 (Piperazine-C), 61.59 (Benzylic-CH2), 66.66 (CH_2_), 127.40, 128.31, 129.19, 130.57 (Ar—C), 155.55 (Triazole C-5), 167.73 (C=S).

### 3. Refinement

All H atoms were positioned geometrically [C—H = 0.93, 0.96, 0.97 or 0.98 Å] and refined using a riding model with *U*_iso_(H) = 1.2 or 1.5(methyl group)*U*_eq_(C).

### Crystal data

**Table:**

C_26_H_37_N_5_S	*Z* = 2
*M**_r_* = 451.67	*F*(000) = 488
Triclinic, *P*1	*D*_x_ = 1.218 Mg m^−^^3^
Hall symbol: -P 1	Cu *K*α radiation, λ = 1.54178 Å
*a* = 10.5618 (2) Å	Cell parameters from 6190 reflections
*b* = 11.2123 (2) Å	θ = 4.0–69.3°
*c* = 11.3084 (2) Å	µ = 1.33 mm^−^^1^
α = 89.974 (1)°	*T* = 296 K
β = 77.619 (1)°	Block, colourless
γ = 70.842 (1)°	0.69 × 0.51 × 0.39 mm
*V* = 1232.03 (4) Å^3^	

### Data collection

**Table:**

Bruker APEXII CCD diffractometer	4319 independent reflections
Radiation source: fine-focus sealed tube	3986 reflections with *I* > 2σ(*I*)
Graphite monochromator	*R*_int_ = 0.025
φ and ω scans	θ_max_ = 67.5°, θ_min_ = 4.0°
Absorption correction: multi-scan (*SADABS*; Bruker, 2009)	*h* = −12→12
*T*_min_ = 0.460, *T*_max_ = 0.626	*k* = −13→13
13169 measured reflections	*l* = −12→10

### Refinement

**Table:**

Refinement on *F*^2^	Secondary atom site location: difference Fourier map
Least-squares matrix: full	Hydrogen site location: inferred from neighbouring sites
*R*[*F*^2^ > 2σ(*F*^2^)] = 0.040	H-atom parameters constrained
*wR*(*F*^2^) = 0.117	*w* = 1/[σ^2^(*F*_o_^2^) + (0.0605*P*)^2^ + 0.2192*P*] where *P* = (*F*_o_^2^ + 2*F*_c_^2^)/3
*S* = 1.08	(Δ/σ)_max_ < 0.001
4319 reflections	Δρ_max_ = 0.22 e Å^−^^3^
291 parameters	Δρ_min_ = −0.19 e Å^−^^3^
0 restraints	Extinction correction: *SHELXTL* (Sheldrick, 2008), Fc^*^=kFc[1+0.001xFc^2^λ^3^/sin(2θ)]^-1/4^
Primary atom site location: structure-invariant direct methods	Extinction coefficient: 0.0033 (5)

### Special details

**Table:**

Geometry. All e.s.d.'s (except the e.s.d. in the dihedral angle between two l.s. planes) are estimated using the full covariance matrix. The cell e.s.d.'s are taken into account individually in the estimation of e.s.d.'s in distances, angles and torsion angles; correlations between e.s.d.'s in cell parameters are only used when they are defined by crystal symmetry. An approximate (isotropic) treatment of cell e.s.d.'s is used for estimating e.s.d.'s involving l.s. planes.
Refinement. Refinement of *F*^2^ against ALL reflections. The weighted *R*-factor *wR* and goodness of fit *S* are based on *F*^2^, conventional *R*-factors *R* are based on *F*, with *F* set to zero for negative *F*^2^. The threshold expression of *F*^2^ > σ(*F*^2^) is used only for calculating *R*-factors(gt) *etc*. and is not relevant to the choice of reflections for refinement. *R*-factors based on *F*^2^ are statistically about twice as large as those based on *F*, and *R*- factors based on ALL data will be even larger.

### Fractional atomic coordinates and isotropic or equivalent isotropic displacement parameters (Å^2^)

**Table:**

	*x*	*y*	*z*	*U*_iso_*/*U*_eq_	
S1	0.22677 (5)	0.18527 (4)	0.13850 (5)	0.06851 (17)	
N1	−0.34170 (12)	0.23635 (12)	0.02812 (11)	0.0489 (3)	
N2	−0.16540 (13)	0.16685 (11)	0.19435 (11)	0.0477 (3)	
N3	−0.03907 (13)	0.29271 (11)	0.26359 (12)	0.0486 (3)	
N4	−0.13337 (13)	0.40759 (11)	0.31410 (11)	0.0485 (3)	
N5	0.06528 (12)	0.42739 (11)	0.22592 (12)	0.0476 (3)	
C1	−0.3188 (2)	0.16668 (18)	−0.27179 (17)	0.0688 (5)	
H1A	−0.2913	0.0807	−0.2598	0.083*	
C2	−0.2848 (2)	0.2056 (2)	−0.38571 (18)	0.0802 (6)	
H2A	−0.2331	0.1456	−0.4498	0.096*	
C3	−0.3260 (2)	0.3322 (2)	−0.40656 (17)	0.0704 (5)	
H3A	−0.3028	0.3579	−0.4841	0.084*	
C4	−0.4019 (2)	0.41987 (18)	−0.31105 (17)	0.0654 (4)	
H4A	−0.4310	0.5056	−0.3240	0.078*	
C5	−0.43506 (17)	0.38159 (17)	−0.19630 (16)	0.0599 (4)	
H5A	−0.4859	0.4419	−0.1323	0.072*	
C6	−0.39358 (15)	0.25397 (16)	−0.17470 (15)	0.0537 (4)	
C7	−0.42804 (17)	0.21280 (18)	−0.04912 (16)	0.0594 (4)	
H7A	−0.5240	0.2581	−0.0122	0.071*	
H7B	−0.4154	0.1231	−0.0542	0.071*	
C8	−0.39368 (16)	0.21841 (17)	0.15458 (15)	0.0567 (4)	
H8A	−0.3900	0.1312	0.1624	0.068*	
H8B	−0.4889	0.2726	0.1813	0.068*	
C9	−0.30821 (16)	0.25001 (16)	0.23323 (15)	0.0560 (4)	
H9A	−0.3131	0.3376	0.2266	0.067*	
H9B	−0.3441	0.2393	0.3175	0.067*	
C10	−0.11059 (15)	0.17746 (15)	0.06677 (14)	0.0524 (4)	
H10A	−0.0178	0.1175	0.0415	0.063*	
H10B	−0.1064	0.2620	0.0557	0.063*	
C11	−0.20000 (16)	0.15135 (17)	−0.01097 (16)	0.0578 (4)	
H11A	−0.1641	0.1624	−0.0951	0.069*	
H11B	−0.1976	0.0643	−0.0054	0.069*	
C12	−0.07720 (17)	0.17719 (14)	0.27078 (15)	0.0517 (4)	
H12A	0.0066	0.1044	0.2506	0.062*	
H12B	−0.1222	0.1729	0.3542	0.062*	
C13	0.08395 (16)	0.30056 (14)	0.20895 (14)	0.0492 (3)	
C14	0.17668 (16)	0.47676 (15)	0.17788 (17)	0.0597 (4)	
H14A	0.2618	0.4197	0.1937	0.072*	
H14B	0.1571	0.5583	0.2201	0.072*	
C15	0.1955 (2)	0.49166 (19)	0.04371 (19)	0.0760 (5)	
H15A	0.2609	0.5345	0.0186	0.114*	
H15B	0.1090	0.5404	0.0264	0.114*	
H15C	0.2287	0.4097	0.0006	0.114*	
C16	−0.06822 (15)	0.48847 (13)	0.28983 (13)	0.0449 (3)	
C17	−0.13467 (15)	0.62762 (13)	0.33107 (14)	0.0464 (3)	
C18	−0.07200 (18)	0.66332 (15)	0.43095 (16)	0.0575 (4)	
H18A	−0.0833	0.6123	0.4991	0.069*	
H18B	0.0257	0.6464	0.3997	0.069*	
C19	−0.1432 (2)	0.80408 (16)	0.47337 (18)	0.0678 (5)	
H19A	−0.1015	0.8264	0.5357	0.081*	
C20	−0.1263 (2)	0.88384 (16)	0.3662 (2)	0.0720 (5)	
H20A	−0.1692	0.9730	0.3933	0.086*	
H20B	−0.0292	0.8678	0.3323	0.086*	
C21	−0.1921 (2)	0.85163 (16)	0.27002 (18)	0.0675 (5)	
H21A	−0.1816	0.9042	0.2018	0.081*	
C22	−0.3438 (2)	0.87674 (18)	0.3226 (2)	0.0792 (6)	
H22A	−0.3861	0.8562	0.2608	0.095*	
H22B	−0.3893	0.9657	0.3495	0.095*	
C23	−0.36012 (19)	0.79624 (18)	0.4289 (2)	0.0737 (5)	
H23A	−0.4583	0.8124	0.4628	0.088*	
C24	−0.28947 (17)	0.65544 (16)	0.38466 (19)	0.0651 (5)	
H24A	−0.3317	0.6342	0.3233	0.078*	
H24B	−0.3014	0.6036	0.4520	0.078*	
C25	−0.2958 (2)	0.82930 (19)	0.5263 (2)	0.0783 (6)	
H25A	−0.3071	0.7784	0.5946	0.094*	
H25B	−0.3409	0.9178	0.5550	0.094*	
C26	−0.12118 (19)	0.71224 (15)	0.22511 (15)	0.0579 (4)	
H26A	−0.0246	0.6969	0.1894	0.069*	
H26B	−0.1627	0.6914	0.1630	0.069*	

### Atomic displacement parameters (Å^2^)

**Table:**

	*U*^11^	*U*^22^	*U*^33^	*U*^12^	*U*^13^	*U*^23^
S1	0.0597 (3)	0.0421 (2)	0.0933 (4)	−0.01144 (18)	−0.0046 (2)	−0.0041 (2)
N1	0.0435 (6)	0.0499 (7)	0.0504 (7)	−0.0141 (5)	−0.0069 (5)	0.0036 (5)
N2	0.0565 (7)	0.0367 (6)	0.0529 (7)	−0.0188 (5)	−0.0140 (5)	0.0067 (5)
N3	0.0568 (7)	0.0347 (6)	0.0569 (7)	−0.0198 (5)	−0.0113 (5)	0.0030 (5)
N4	0.0538 (7)	0.0387 (6)	0.0552 (7)	−0.0200 (5)	−0.0097 (5)	0.0024 (5)
N5	0.0475 (6)	0.0368 (6)	0.0607 (8)	−0.0178 (5)	−0.0112 (5)	0.0029 (5)
C1	0.0811 (12)	0.0559 (10)	0.0660 (11)	−0.0199 (9)	−0.0143 (9)	−0.0018 (8)
C2	0.1022 (15)	0.0737 (13)	0.0568 (11)	−0.0282 (11)	−0.0035 (10)	−0.0116 (9)
C3	0.0840 (12)	0.0775 (13)	0.0564 (10)	−0.0368 (10)	−0.0149 (9)	0.0063 (8)
C4	0.0700 (11)	0.0592 (10)	0.0710 (12)	−0.0235 (9)	−0.0213 (9)	0.0071 (8)
C5	0.0556 (9)	0.0596 (10)	0.0609 (10)	−0.0164 (8)	−0.0103 (7)	−0.0049 (7)
C6	0.0463 (8)	0.0607 (9)	0.0573 (9)	−0.0200 (7)	−0.0152 (6)	0.0018 (7)
C7	0.0519 (8)	0.0691 (11)	0.0614 (10)	−0.0258 (8)	−0.0132 (7)	0.0068 (8)
C8	0.0502 (8)	0.0644 (10)	0.0552 (9)	−0.0231 (7)	−0.0052 (7)	0.0060 (7)
C9	0.0550 (9)	0.0611 (10)	0.0500 (9)	−0.0230 (7)	−0.0024 (7)	0.0006 (7)
C10	0.0444 (7)	0.0501 (8)	0.0556 (9)	−0.0092 (6)	−0.0070 (6)	0.0005 (6)
C11	0.0489 (8)	0.0602 (10)	0.0550 (9)	−0.0087 (7)	−0.0072 (7)	−0.0058 (7)
C12	0.0667 (9)	0.0372 (7)	0.0594 (9)	−0.0247 (7)	−0.0200 (7)	0.0123 (6)
C13	0.0552 (8)	0.0384 (7)	0.0572 (9)	−0.0184 (6)	−0.0151 (7)	0.0047 (6)
C14	0.0478 (8)	0.0450 (8)	0.0874 (12)	−0.0212 (7)	−0.0089 (8)	0.0019 (7)
C15	0.0716 (11)	0.0614 (11)	0.0862 (14)	−0.0250 (9)	0.0045 (10)	0.0112 (9)
C16	0.0489 (7)	0.0374 (7)	0.0520 (8)	−0.0176 (6)	−0.0138 (6)	0.0041 (6)
C17	0.0483 (8)	0.0351 (7)	0.0580 (9)	−0.0152 (6)	−0.0145 (6)	0.0027 (6)
C18	0.0659 (10)	0.0455 (8)	0.0632 (10)	−0.0149 (7)	−0.0251 (8)	0.0019 (7)
C19	0.0816 (12)	0.0502 (9)	0.0746 (12)	−0.0187 (9)	−0.0292 (9)	−0.0094 (8)
C20	0.0787 (12)	0.0377 (8)	0.0997 (15)	−0.0230 (8)	−0.0151 (10)	−0.0030 (8)
C21	0.0830 (12)	0.0408 (9)	0.0741 (12)	−0.0137 (8)	−0.0196 (9)	0.0138 (8)
C22	0.0741 (12)	0.0502 (10)	0.1061 (16)	−0.0029 (9)	−0.0337 (11)	0.0037 (10)
C23	0.0524 (9)	0.0517 (10)	0.1044 (15)	−0.0100 (8)	−0.0034 (9)	−0.0068 (9)
C24	0.0525 (9)	0.0500 (9)	0.0910 (13)	−0.0194 (7)	−0.0088 (8)	−0.0014 (8)
C25	0.0895 (14)	0.0522 (10)	0.0762 (13)	−0.0130 (10)	−0.0006 (10)	−0.0086 (8)
C26	0.0698 (10)	0.0446 (8)	0.0587 (10)	−0.0164 (7)	−0.0180 (8)	0.0071 (7)

### Geometric parameters (Å, º)

**Table:**

S1—C13	1.6694 (16)	C11—H11B	0.9700
N1—C8	1.457 (2)	C12—H12A	0.9700
N1—C11	1.4574 (19)	C12—H12B	0.9700
N1—C7	1.471 (2)	C14—C15	1.504 (3)
N2—C12	1.4299 (19)	C14—H14A	0.9700
N2—C10	1.455 (2)	C14—H14B	0.9700
N2—C9	1.460 (2)	C15—H15A	0.9600
N3—C13	1.345 (2)	C15—H15B	0.9600
N3—N4	1.3756 (17)	C15—H15C	0.9600
N3—C12	1.4746 (18)	C16—C17	1.5119 (19)
N4—C16	1.3037 (18)	C17—C18	1.539 (2)
N5—C13	1.3777 (18)	C17—C26	1.540 (2)
N5—C16	1.3817 (19)	C17—C24	1.542 (2)
N5—C14	1.463 (2)	C18—C19	1.534 (2)
C1—C2	1.372 (3)	C18—H18A	0.9700
C1—C6	1.382 (2)	C18—H18B	0.9700
C1—H1A	0.9300	C19—C20	1.520 (3)
C2—C3	1.378 (3)	C19—C25	1.523 (3)
C2—H2A	0.9300	C19—H19A	0.9800
C3—C4	1.374 (3)	C20—C21	1.512 (3)
C3—H3A	0.9300	C20—H20A	0.9700
C4—C5	1.376 (3)	C20—H20B	0.9700
C4—H4A	0.9300	C21—C22	1.515 (3)
C5—C6	1.390 (2)	C21—C26	1.527 (2)
C5—H5A	0.9300	C21—H21A	0.9800
C6—C7	1.500 (2)	C22—C23	1.518 (3)
C7—H7A	0.9700	C22—H22A	0.9700
C7—H7B	0.9700	C22—H22B	0.9700
C8—C9	1.512 (2)	C23—C25	1.516 (3)
C8—H8A	0.9700	C23—C24	1.539 (2)
C8—H8B	0.9700	C23—H23A	0.9800
C9—H9A	0.9700	C24—H24A	0.9700
C9—H9B	0.9700	C24—H24B	0.9700
C10—C11	1.513 (2)	C25—H25A	0.9700
C10—H10A	0.9700	C25—H25B	0.9700
C10—H10B	0.9700	C26—H26A	0.9700
C11—H11A	0.9700	C26—H26B	0.9700
			
C8—N1—C11	108.65 (12)	N5—C14—H14B	109.1
C8—N1—C7	110.90 (12)	C15—C14—H14B	109.1
C11—N1—C7	110.90 (13)	H14A—C14—H14B	107.8
C12—N2—C10	113.10 (12)	C14—C15—H15A	109.5
C12—N2—C9	114.96 (12)	C14—C15—H15B	109.5
C10—N2—C9	110.90 (12)	H15A—C15—H15B	109.5
C13—N3—N4	112.98 (11)	C14—C15—H15C	109.5
C13—N3—C12	126.34 (13)	H15A—C15—H15C	109.5
N4—N3—C12	120.67 (12)	H15B—C15—H15C	109.5
C16—N4—N3	104.98 (12)	N4—C16—N5	110.22 (12)
C13—N5—C16	108.38 (12)	N4—C16—C17	122.45 (13)
C13—N5—C14	120.92 (13)	N5—C16—C17	127.32 (12)
C16—N5—C14	130.70 (12)	C16—C17—C18	110.91 (12)
C2—C1—C6	120.65 (18)	C16—C17—C26	111.94 (13)
C2—C1—H1A	119.7	C18—C17—C26	109.46 (13)
C6—C1—H1A	119.7	C16—C17—C24	108.86 (12)
C1—C2—C3	120.97 (18)	C18—C17—C24	107.90 (14)
C1—C2—H2A	119.5	C26—C17—C24	107.63 (13)
C3—C2—H2A	119.5	C19—C18—C17	109.97 (13)
C4—C3—C2	118.94 (18)	C19—C18—H18A	109.7
C4—C3—H3A	120.5	C17—C18—H18A	109.7
C2—C3—H3A	120.5	C19—C18—H18B	109.7
C3—C4—C5	120.39 (18)	C17—C18—H18B	109.7
C3—C4—H4A	119.8	H18A—C18—H18B	108.2
C5—C4—H4A	119.8	C20—C19—C25	109.56 (16)
C4—C5—C6	120.91 (16)	C20—C19—C18	109.55 (15)
C4—C5—H5A	119.5	C25—C19—C18	109.49 (16)
C6—C5—H5A	119.5	C20—C19—H19A	109.4
C1—C6—C5	118.12 (16)	C25—C19—H19A	109.4
C1—C6—C7	121.14 (16)	C18—C19—H19A	109.4
C5—C6—C7	120.73 (15)	C21—C20—C19	109.95 (15)
N1—C7—C6	112.00 (13)	C21—C20—H20A	109.7
N1—C7—H7A	109.2	C19—C20—H20A	109.7
C6—C7—H7A	109.2	C21—C20—H20B	109.7
N1—C7—H7B	109.2	C19—C20—H20B	109.7
C6—C7—H7B	109.2	H20A—C20—H20B	108.2
H7A—C7—H7B	107.9	C20—C21—C22	109.80 (17)
N1—C8—C9	110.02 (13)	C20—C21—C26	109.38 (14)
N1—C8—H8A	109.7	C22—C21—C26	109.75 (16)
C9—C8—H8A	109.7	C20—C21—H21A	109.3
N1—C8—H8B	109.7	C22—C21—H21A	109.3
C9—C8—H8B	109.7	C26—C21—H21A	109.3
H8A—C8—H8B	108.2	C21—C22—C23	109.30 (15)
N2—C9—C8	109.63 (13)	C21—C22—H22A	109.8
N2—C9—H9A	109.7	C23—C22—H22A	109.8
C8—C9—H9A	109.7	C21—C22—H22B	109.8
N2—C9—H9B	109.7	C23—C22—H22B	109.8
C8—C9—H9B	109.7	H22A—C22—H22B	108.3
H9A—C9—H9B	108.2	C25—C23—C22	109.94 (17)
N2—C10—C11	110.69 (13)	C25—C23—C24	109.75 (16)
N2—C10—H10A	109.5	C22—C23—C24	109.33 (17)
C11—C10—H10A	109.5	C25—C23—H23A	109.3
N2—C10—H10B	109.5	C22—C23—H23A	109.3
C11—C10—H10B	109.5	C24—C23—H23A	109.3
H10A—C10—H10B	108.1	C23—C24—C17	110.23 (14)
N1—C11—C10	110.90 (13)	C23—C24—H24A	109.6
N1—C11—H11A	109.5	C17—C24—H24A	109.6
C10—C11—H11A	109.5	C23—C24—H24B	109.6
N1—C11—H11B	109.5	C17—C24—H24B	109.6
C10—C11—H11B	109.5	H24A—C24—H24B	108.1
H11A—C11—H11B	108.0	C23—C25—C19	109.13 (16)
N2—C12—N3	116.03 (12)	C23—C25—H25A	109.9
N2—C12—H12A	108.3	C19—C25—H25A	109.9
N3—C12—H12A	108.3	C23—C25—H25B	109.9
N2—C12—H12B	108.3	C19—C25—H25B	109.9
N3—C12—H12B	108.3	H25A—C25—H25B	108.3
H12A—C12—H12B	107.4	C21—C26—C17	110.28 (14)
N3—C13—N5	103.43 (13)	C21—C26—H26A	109.6
N3—C13—S1	128.88 (11)	C17—C26—H26A	109.6
N5—C13—S1	127.69 (12)	C21—C26—H26B	109.6
N5—C14—C15	112.60 (15)	C17—C26—H26B	109.6
N5—C14—H14A	109.1	H26A—C26—H26B	108.1
C15—C14—H14A	109.1		
			
C13—N3—N4—C16	0.40 (16)	N3—N4—C16—C17	−179.14 (13)
C12—N3—N4—C16	−178.80 (13)	C13—N5—C16—N4	0.30 (17)
C6—C1—C2—C3	−1.0 (3)	C14—N5—C16—N4	179.45 (15)
C1—C2—C3—C4	0.2 (3)	C13—N5—C16—C17	178.95 (14)
C2—C3—C4—C5	0.5 (3)	C14—N5—C16—C17	−1.9 (3)
C3—C4—C5—C6	−0.5 (3)	N4—C16—C17—C18	109.96 (16)
C2—C1—C6—C5	1.1 (3)	N5—C16—C17—C18	−68.55 (19)
C2—C1—C6—C7	−178.28 (18)	N4—C16—C17—C26	−127.47 (15)
C4—C5—C6—C1	−0.3 (3)	N5—C16—C17—C26	54.03 (19)
C4—C5—C6—C7	179.02 (15)	N4—C16—C17—C24	−8.6 (2)
C8—N1—C7—C6	169.20 (14)	N5—C16—C17—C24	172.88 (15)
C11—N1—C7—C6	−69.99 (18)	C16—C17—C18—C19	−178.70 (14)
C1—C6—C7—N1	104.19 (18)	C26—C17—C18—C19	57.30 (18)
C5—C6—C7—N1	−75.16 (19)	C24—C17—C18—C19	−59.55 (18)
C11—N1—C8—C9	60.95 (17)	C17—C18—C19—C20	−58.9 (2)
C7—N1—C8—C9	−176.91 (14)	C17—C18—C19—C25	61.3 (2)
C12—N2—C9—C8	−172.94 (12)	C25—C19—C20—C21	−59.28 (19)
C10—N2—C9—C8	57.20 (16)	C18—C19—C20—C21	60.8 (2)
N1—C8—C9—N2	−60.32 (17)	C19—C20—C21—C22	59.44 (19)
C12—N2—C10—C11	173.70 (12)	C19—C20—C21—C26	−61.1 (2)
C9—N2—C10—C11	−55.46 (16)	C20—C21—C22—C23	−59.6 (2)
C8—N1—C11—C10	−59.08 (18)	C26—C21—C22—C23	60.7 (2)
C7—N1—C11—C10	178.79 (13)	C21—C22—C23—C25	60.2 (2)
N2—C10—C11—N1	56.68 (18)	C21—C22—C23—C24	−60.4 (2)
C10—N2—C12—N3	55.67 (17)	C25—C23—C24—C17	−60.1 (2)
C9—N2—C12—N3	−73.11 (17)	C22—C23—C24—C17	60.5 (2)
C13—N3—C12—N2	−105.86 (17)	C16—C17—C24—C23	179.36 (15)
N4—N3—C12—N2	73.21 (18)	C18—C17—C24—C23	58.92 (19)
N4—N3—C13—N5	−0.21 (16)	C26—C17—C24—C23	−59.11 (19)
C12—N3—C13—N5	178.92 (13)	C22—C23—C25—C19	−60.1 (2)
N4—N3—C13—S1	179.12 (11)	C24—C23—C25—C19	60.2 (2)
C12—N3—C13—S1	−1.7 (2)	C20—C19—C25—C23	59.33 (19)
C16—N5—C13—N3	−0.04 (16)	C18—C19—C25—C23	−60.8 (2)
C14—N5—C13—N3	−179.29 (14)	C20—C21—C26—C17	59.6 (2)
C16—N5—C13—S1	−179.39 (12)	C22—C21—C26—C17	−60.88 (19)
C14—N5—C13—S1	1.4 (2)	C16—C17—C26—C21	178.81 (13)
C13—N5—C14—C15	77.74 (18)	C18—C17—C26—C21	−57.80 (18)
C16—N5—C14—C15	−101.32 (19)	C24—C17—C26—C21	59.22 (18)
N3—N4—C16—N5	−0.41 (15)		
